# Supplementary material for: 500-year paleoclimate record inferred from Greenland Juniper wood contextualizes current climate warming
Source: Nat Commun. 2025 Nov 26;16:11665. doi: 10.1038/s41467-025-66842-1 (PMC12749314; doi:10.1038/s41467-025-66842-1)
Supplement: Supplementary file 1 — Supplementary Information [file 41467_2025_66842_MOESM1_ESM.pdf]

SUPPLEMENTARY INFORMATION FOR

500-year paleoclimate record inferred from Greenland Juniper wood contextualizes current climate warming

Magdalena Opała-Owczarek[1*], Ulf Büntgen[2,3,4], Piotr Owczarek[5], Christian Lange[6]

[1]Institute of Earth Sciences, Faculty of Natural Sciences, University of Silesia in Katowice, Sosnowiec, Poland. [2]Department of Geography, University of Cambridge, Cambridge CB2 3EN, United Kingdom. [3]Global Change Research Institute (CzechGlobe), Czech Academy of Sciences, 603 00 Brno, Czech Republic. [4]Department of Geography, Faculty of Science, Masaryk University, 613 00 Brno, Czech Republic. [5]Institute of Geography and Regional Development, University of Wroclaw, Wroclaw, Poland. [6]Natural History Museum, Copenhagen, Denmark.

SUPPLEMENTARY TABLES

Supplementary Table 1. List of herbaria samples, with dendrochronological dating (the starting and ending calendar years), the number of rings and the original collector and date of collection, in descending order of the number of rings for dendrochronologically dated and undated samples, respectively.

No	Lab Code	Crossdating		No of Years	Original counting	the collector's name	Date of sampling	Sampling Location
		Starting year	Ending year					
1	8104a	1536	1889	354	367	N.Hartz	1889	Tasermiut
2	8064	1538	1885	348	351	C.Lytzen	1886	Tasermiut
3	8145	1536	1874	339	-	unknown	unknown	Tasermiut
4	8104b	1536	1874	338	-	N.Hartz	-	Tasermiut
5	8087	1617	1874	258	342	C.Lytzen	unknown	Igaliku
6	8117po	1687	1894	208	252	C.Lytzen	unknown	Igaliku
7	8091	1688	1886	199	199	C.Lytzen	1886	Tasermiut
8	8116	1728	1902	175	200	C.Lytzen	unknown	Igaliku
9	8086	1727	1886	160	-	C.Lytzen	1886	Tasermiut
10	8113	1732	1889	158	179	N.Hartz	1889	Tasermiut
11	8093b	1735	1887	153	-	C.Lytzen	1887	Igaliku
12	8107	1735	1886	152	c. 150	C.Lytzen	1888	Julianehaab Fjord
13	8081	1757	1886	130	-	C.Lytzen	1886	Qaqortoq
14	8112	1762	1885	124	135	C.Lytzen	1886	Qaqortoq
15	8093f	1790	1887	97	-	C.Lytzen	1887	Igaliku
16	8093a	1803	1876	74	-	C.Lytzen	1887	Igaliku
17	8093d	1805	1877	73	-	C.Lytzen	1887	Igaliku
18	8110	1814	1884	71	-	C.Lytzen	1886	Qaqortoq
19	8105	-	-	251	c.309?	C.Lytzen	1886	Alluitsup
20	8106	-	-	251	-	C.Lytzen	1886	Alluitsup
21	8090	-	-	235	c.287	C.Lytzen	unknown	Igaliku
22	8067	-	-	233	c.235	N.Hartz	1889	Unartoq
23	8115	-	-	160	158	-	-	Tunulliarfik Fjord
24	8093c	-	-	144	-	C.Lytzen	1887	Igaliku
25	8085	-	-	140	139	N.Hartz	1889	?
26	8094	-	-	80	83	-	24 Jul 1888	Arsuk Fjord
27	8093f	-	-	73	-	C.Lytzen	1887	Igaliku
28	8093e	-	-	68	-	C.Lytzen	1887	Igaliku
29	8111	-	-	27	-	C.Lytzen	1886	Qaqortoq
30	8108	-	-	?	c.380	C.Lytzen	1886	Tasermiut
31	8109	-	-	?	c.210	C.Lytzen	1886	Qaqortoq
32	8095	-	-	-	-	-	3 Jul 1888	Arsuk Fjord
33	8088	-	-	-	Over 200	-	17 Jun 1888	Tindingen
34	8083	-	-	-	241	-	2 Sep 1888	Arsuk Fjord
35	8082	-	-	-	-	C.Lytzen	1886	Tasermiut

Supplementary Table 2. List of ten oldest individuals collected at each sites, with dendrochronological dating (the starting and ending calendar years), the number of rings in descending order of the number of rings.

No	Lab Code	Crossdating		No of Years	Sampling Location
		Starting year	Ending year		
1	n3z-02-3	1694	2022	329	Tasiusaq
2	n3z-03	1796	2022	227	Tasiusaq
3	n3z-08	1818	2023	206	Tasiusaq
4	n3s-02	1541	1744	204	Tasiusaq
5	n3z-10	1833	2023	191	Tasiusaq
6	n3z-01	1850	2022	173	Tasiusaq
7	n3z-07	1861	2023	163	Tasiusaq
8	n3z-05	1875	2023	149	Tasiusaq
9	n3z-09	1875	2023	149	Tasiusaq
10	n3s-12	1764	1906	143	Tasiusaq
1	n2z-05	1657	2023	367	Sermiat
2	n2s-11*	-	-	320	Sermiat
3	n2z-01	1716	2023	308	Sermiat
4	n2s-01*	-	-	297	Sermiat
5	f2z-04	1735	2023	289	Sermiat
6	n2s-10*	-	-	285	Sermiat
7	n2z-03	1748	2022	275	Sermiat
8	n2z-02	1879	2023	145	Sermiat
9	n2s-03sk	1878	2011	134	Sermiat
10	n2z-10	1758	1890	133	Sermiat
1	n1z-04	1821	2023	203	Narsarsuaq
2	n1z-06	1866	2008	143	Narsarsuaq
3	n1z-05	1889	2023	135	Narsarsuaq
4	n1z-01	1889	2025	135	Narsarsuaq
5	n1z-00	1893	2023	131	Narsarsuaq
6	n1z-02	1975	2023	49	Narsarsuaq
7	n1z-07	1978	2023	46	Narsarsuaq
8	fd-01	1980	2023	44	Narsarsuaq
9	fd-02	1981	2023	43	Narsarsuaq
10	n1z-03	1984	2023	40	Narsarsuaq

*note that in some cases dendrochronological dating was not possible and only number of rings is given
