# Supplementary material for: 500-year paleoclimate record inferred from Greenland Juniper wood contextualizes current climate warming
Source: Nat Commun. 2025 Nov 26;16:11665. doi: 10.1038/s41467-025-66842-1 (PMC12749314; doi:10.1038/s41467-025-66842-1)
Supplement: Supplementary file 2 — Reporting Summary [file 41467_2025_66842_MOESM2_ESM.pdf]

nature portfolio

Corresponding author(s): Magdalena Opała-Owczarek

Last updated by author(s): Oct 26, 2025

Reporting Summary

Nature Portfolio wishes to improve the reproducibility of the work that we publish. This form provides structure for consistency and transparency in reporting. For further information on Nature Portfolio policies, see our Editorial Policies and the Editorial Policy Checklist.

Statistics

For all statistical analyses, confirm that the following items are present in the figure legend, table legend, main text, or Methods section.

n/a	Confirmed	
☐	☒	The exact sample size (*n*) for each experimental group/condition, given as a discrete number and unit of measurement
☐	☒	A statement on whether measurements were taken from distinct samples or whether the same sample was measured repeatedly
☐	☒	The statistical test(s) used AND whether they are one- or two-sided<br>*Only common tests should be described solely by name; describe more complex techniques in the Methods section.*
☐	☒	A description of all covariates tested
☒	☐	A description of any assumptions or corrections, such as tests of normality and adjustment for multiple comparisons
☐	☒	A full description of the statistical parameters including central tendency (e.g. means) or other basic estimates (e.g. regression coefficient) AND variation (e.g. standard deviation) or associated estimates of uncertainty (e.g. confidence intervals)
☐	☒	For null hypothesis testing, the test statistic (e.g. *F*, *t*, *r*) with confidence intervals, effect sizes, degrees of freedom and *P* value noted<br>*Give P values as exact values whenever suitable.*
☒	☐	For Bayesian analysis, information on the choice of priors and Markov chain Monte Carlo settings
☒	☐	For hierarchical and complex designs, identification of the appropriate level for tests and full reporting of outcomes
☐	☒	Estimates of effect sizes (e.g. Cohen's *d*, Pearson's *r*), indicating how they were calculated

Our web collection on statistics for biologists contains articles on many of the points above.

Software and code

Policy information about availability of computer code

Data collection	Leica LAS-X for the Leica Microscope for image acquisition and PTGui for merging of multiple microscopic images of wood sections
Data analysis	Measurements of the width of annual growth rings were made on the basis of digital images using WinDENDRO software. COFECHA software was used for dating and measurement quality check; dplR R package for detrending; treeclim R package for Pearson's correlation and bootstrapped correlation analyses, moving correlation, R2, CE, RE. Superposed epoch analysis was calculated using SEA computation from the dplR package. No custom algorithms were used. Code availability: Statistical analysis in this study was performed with publicly available packages in R (version 4.4.1).

For manuscripts utilizing custom algorithms or software that are central to the research but not yet described in published literature, software must be made available to editors and reviewers. We strongly encourage code deposition in a community repository (e.g. GitHub). See the Nature Portfolio guidelines for submitting code & software for further information.

Data

Policy information about availability of data

All manuscripts must include a data availability statement. This statement should provide the following information, where applicable:

- Accession codes, unique identifiers, or web links for publicly available datasets
- A description of any restrictions on data availability
- For clinical datasets or third party data, please ensure that the statement adheres to our policy

Main source data are provided in the Source Data file. Additional data related to these studies may be made available by the authors upon reasonable request, as this data is part of an ongoing project. Other datasets used for this research were derived from the public domain resources. Meteorological data were obtained from the Danish Meteorological Institute (DMI). Satellite-derived Arctic sea ice extent data from 1978–2023 were obtained from the National Snow and Ice Data Centre (NSIDC) and from compilations prepared by Connolly et al. (2017) available as Supplemental material at https://www.tandfonline.com/doi/figure/10.1080/02626667.2017.1324974?scroll=top&needAccess=true.Ice-core inferred non-sea-salt sulphur records from Greenland compiled by Sigl et al. (2015) is available as Source data to Fig. 2 at https://www.nature.com/articles/nature14565. The palaeoclimate data used for comparisons are sourced from primary publications with references provided alongside the relevant figures.

Research involving human participants, their data, or biological material

Policy information about studies with human participants or human data. See also policy information about sex, gender (identity/presentation), and sexual orientation and race, ethnicity and racism.

Reporting on sex and gender	This is not applicable to the study.
Reporting on race, ethnicity, or other socially relevant groupings	This is not applicable to the study.
Population characteristics	This is not applicable to the study.
Recruitment	This is not applicable to the study.
Ethics oversight	This is not applicable to the study.

Note that full information on the approval of the study protocol must also be provided in the manuscript.

Field-specific reporting

Please select the one below that is the best fit for your research. If you are not sure, read the appropriate sections before making your selection.

☐ Life sciences ☐ Behavioural & social sciences ☒ Ecological, evolutionary & environmental sciences

For a reference copy of the document with all sections, see nature.com/documents/nr-reporting-summary-flat.pdf

Ecological, evolutionary & environmental sciences study design

All studies must disclose on these points even when the disclosure is negative.

Study description	We study juniper wood from southern Greenland as a source of information on temperature variability. For the first time, an incremental chronology covering the last 500 years has been constructed for Greenland. We hypothesise that this chronology can be used to reconstruct pre-instrumental climate conditions in Greenlandic terrestrial areas outside the ice sheet.
Research sample	To establish a 500-year chronology, we used the only long-lived species found in Greenland: Juniperus communis L. subsp. nana Syme. of the Cupressaceae. We used samples from living, dry and historical specimens from the herbarium of Greenland Herbarium in Copenhagen, housed at the Natural History Museum of Denmark. The oldest living shrub contained 368 annual rings, which, in combination with historical and dry wood samples up to 354 years old, made it possible to construct a reliable, well replicated 498-year annual ring chronology (1526-2023 A.D.).
Sampling strategy	The labels on the herbarium specimens were used to identify the exact locations where they were collected at the turn of the 19th and 20th centuries. Parts of both living and dry specimens were then collected from the same area in 2023. Dendrochronological studies require the collection of at least 10 well-matched samples from a single site. The data obtained are characterised by sufficiently high statistical indices in the constructed chronology (mean r, rbar, eps). Due to the natural environment in southern Greenland, it is not possible to collect hundreds of samples from a homogeneous area. A total of 127 samples were analysed, 92 from the field and 35 from the herbarium. All available historical material was analysed.
Data collection	Juniper samples with a thickness of 2-3 cm were collected in the field. Measurements of increment widths and anatomical analyses were made from microscopic photographs or scans of the specimens. Historical data were provided on the basis of the herbarium's

	collection permit. High-resolution images of cross sections of historical specimens were taken by Christian Lange. Microscopic preparations were made by Piotr Owczarek. All measurements were made by Magdalena Opała-Owczarek.
Timing and spatial scale	Start and end of work in the herbarium was: 23-27 August 2021; start and end of field work was: 18-30 July 2023.
Data exclusions	Some of the data used in the dendrochronological analyses were excluded from further analysis due to: the presence of anatomical anomalies (wounds, reaction wood), inconsistency of the measurement sequence with the population mean. This is standard practice for woody plants with very narrow rings, such as Arctic shrubs and dwarf shrubs.
Reproducibility	Disks and microscope slides are stored both as raw data and in digital format, thus ensuring reproducibility of measurements. In addition, the sampling locations in the field are indicated, as well as the storage location of the historical discs.
Randomization	In general, sampling was random, taking into account the location of the plant to ensure site homogeneity and to exclude the influence of abiotic factors on growth such as mass movements.
Blinding	Blinding was not relevant to this study. As the number of specimens growing in the study area was limited and those whose growth was not disturbed by geomorphological factors were selected for sampling.

Did the study involve field work? ☒ Yes ☐ No

Field work, collection and transport

Field conditions	South Greenland has variable climatic conditions, with a predominance of fog, high cloud cover and relatively low temperatures. In addition, strong winds and large waves make access to the research sites difficult. Given the meteorological conditions and the morphologically varied terrain, sampling was only possible in the second half of July and only on days when it was safe to sail.
Location	The sites selected for this study are located in southern Greenland (60–61°30′N, 44°30–47°W).
Access & import/export	The historical juniper specimens originate from the Herbarium at the Natural History Museum, University of Copenhagen, Denmark. Permission to conduct the museum query was granted by Collection Manager Olof Ryding and Collection Assistant Karen Bach in 2021. No destructive sampling was performed. The research used high-resolution photographs of juniper cross-sections collected during historical polar expeditions, which were made available by the collection manager of the Greenland Herbarium at the Natural History Museum of Denmark in Copenhagen. The research in southern Greenland was conducted outside the protected area, using a plant species that is common in the area and not protected. The studied species is not listed under the Washington Convention/ CITES.
Disturbance	No destructive sampling was performed on herbarium samples. In order to minimize environmental impact, a minimal number of samples were taken in the field, focusing on dry specimens. In the case of living speciments a sample was taken from the main shoot, preserving the rest of the plant.

Reporting for specific materials, systems and methods

We require information from authors about some types of materials, experimental systems and methods used in many studies. Here, indicate whether each material, system or method listed is relevant to your study. If you are not sure if a list item applies to your research, read the appropriate section before selecting a response.

Materials & experimental systems

n/a	Involved in the study	
☒	☐	Antibodies
☒	☐	Eukaryotic cell lines
☒	☐	Palaeontology and archaeology
☒	☐	Animals and other organisms
☒	☐	Clinical data
☒	☐	Dual use research of concern
☐	☒	Plants

Methods

n/a	Involved in the study	
☒	☐	ChIP-seq
☒	☐	Flow cytometry
☒	☐	MRI-based neuroimaging

Plants

Seed stocks	This is not applicable to the study.
Novel plant genotypes	This is not applicable to the study.
Authentication	This is not applicable to the study.
